# Minimal residual disease in gastroesophageal adenocarcinoma: the search for the invisible

**DOI:** 10.1016/j.esmoop.2022.100547

**Published:** 2022-07-15

**Authors:** N. Tarazona, F. Gimeno-Valiente, A. Cervantes

**Affiliations:** 1 Department of Medical Oncology, INCLIVA Biomedical Research Institute, University of Valencia, Madrid, Spain; 2 Instituto de Salud Carlos III, CIBERONC, Madrid, Spain; 3 Cancer Evolution and Genome Instability Laboratory, University College London Cancer Institute, London, UK

Gastroesophageal adenocarcinoma (GEA) is a lethal disease, with fewer than 35% of patients surviving beyond 5 years despite an optimal neoadjuvant chemotherapy followed by surgery with curative intent.^1^ In an effort to improve patient outcomes, genomic profiling has improved the identification of oncogenic alterations, which could guide a molecular-based approach.^2^ Despite the development of targeted therapies against driver genes such as *MET*, *FGFR2*, *EGFR* and *ERBB2*,^3^, ^4^, ^5^, ^6^ only trastuzumab against ERBB2-amplified tumors has been regulatory approved.^6^ The high intratumor heterogeneity (ITH) could be considered one of the major determinants of the poor success of targeted therapies in GEA. ITH found both within the primary and between primary and metastatic tumors shows the relevance of the clonal and dynamic molecular approach to distinguish target alterations.^7^ Therefore, data indicate that current tissue sampling practices for biomarker testing do not effectively guide personalized medicine in this disease.

In this context, liquid biopsy has emerged as a potential tool to overcome the heterogeneity barrier. Circulating tumor DNA (ctDNA) analysis allows capturing the whole tumor genetic profile present within a patient, unlike tissue biopsies which are limited by the partial analysis achievable from only one tumor region. In the last few years, ctDNA analysis has been proposed as a noninvasive real-time biomarker to provide prognostic and predictive information for monitoring treatment. The presence of ctDNA in plasma following surgical resection has been strongly associated with a high risk of recurrence across tumor types.^8^, ^9^, ^10^, ^11^ Likewise, the evidence for the prognostic value of ctDNA in patients with operable esophageal adenocarcinoma (EAC) to detect minimal residual disease (MRD) has been demonstrated in some cohorts.^7^^,^^12^, ^13^, ^14^

In discordant primary and metastatic lesions, several studies demonstrated high concordance for targetable alterations in metastatic tissue and ctDNA samples,^7^ suggesting the potential for ctDNA profiling to enhance selection of therapy. In advanced gastrointestinal cancer, the presence of specific mutations in plasma can be used to select patients for molecular-matched therapy^15^ and to detect the emergence of resistant clones suitable for subsequent lines of treatment.^16^ Liquid biopsy using ctDNA may ultimately provide a more accurate genomic landscape of tumor tissue, potentially reducing the need for costly and invasive metastatic biopsies.

Currently, there are two approaches for assessing MRD using ctDNA: one that is informed by genomic sequencing of the primary tumor (tumor-informed) and another that is uninformed by the mutations in the primary tumor (tumor-agnostic). However, what is the ideal assay to monitor MRD remains unanswered.

In this issue of *ESMO Open*, Bonazzi and colleagues^17^ describe the results from a commercially available pancancer mutation panel evaluated in both ctDNA and tumor samples in a single cohort from resected EAC (*n* = 57). Moreover, whole-genome and whole-exome sequencing was carried out on the primary tissue in a subset of patients (*n* = 18). The main objectives of the study were to determine: (i) the prognostic value of pre- and post-operative ctDNA analysis using both a tumor-agnostic and a tumor-informed approach, (ii) whether ctDNA profile is reflective of ITH, and (iii) the ability of serial ctDNA monitoring to detect recurrence during follow-up.

Plasma ctDNA detectable at baseline in patients with resectable EAC was not associated with worse survival. The detection, however, of ctDNA after treatment, either after neoadjuvant therapy or surgery, was significantly associated with worse disease-specific survival using both the tumor-agnostic (*P* = 0.0130) and the tumor-informed (*P* = 0.0007) approaches. ctDNA is considered as a surrogate endpoint for MRD that is correlated to the definitive endpoint progression-free survival (PFS) in adjuvant trials.^18^^,^^19^ This study favored a tumor-informed approach over a tumor-agnostic test to find a worse PFS (median survival 9.3 versus 60 months, *P =* 0.0311) in ctDNA-positive versus ctDNA-negative patients. Nevertheless, it is remarkable that 66% of patients without detectable ctDNA after surgery died from disease at 40 months suggesting a low sensitivity for both tests. A potential explanation of the relatively high recurrence rate among those patients is the use of a non-EAC-specific gene panel, which could fail to detect EAC-specific mutations.^12^ On the other hand, localized EAC has been shown to be a low ctDNA shedding tumor, which would limit the power of detection of MRD through ctDNA despite using tumor-specific panels.^14^

This research suggests that a tumor-informed approach helps in determining MRD with both high sensitivity and specificity as it leads to the identification of tumor-specific variants at a very low variant allele frequency (VAF) of 0.01%. Besides tumor-naïve tests being less sensitive, they are able to detect tumor-specific variants for a VAF between 0.1% and 1%. Overall, carrying out the same depth molecular analyses for both primary tumor and matched PL plasma samples increases the possibility of monitoring true tumor-derived clonal variants.^10^^,^^20^ A multidisciplinary workshop organized to discuss routine implementation of liquid biopsies in cancer management concluded that tumor-informed assays will most likely provide better clinical utility than tumor-agnostic tests, yet at a higher cost.^21^ Other approaches for tracking MRD that are currently also being studied, such as analyzing ctDNA methylation, need further validation before they can be used in a clinical setting.^22^

The authors also sequenced multi-region primary tumor samples in a subset of 17 patients reporting that 94% of them harbored at least one mutation. These data confirm that sequencing multiple tumor regions increases the sensitivity to detect tumor variants. Nevertheless, concordance between primary tumor and ctDNA variants at diagnosis was as low as 22% of patients sequenced across several platforms. In this study, ctDNA testing did not reflect the genomic profile of different tumor locations sequenced. This effect should be assessed through the cancer cell fraction (CCF), which would allow understanding the molecular aberrations belonging to each of the possible clones within each region.^23^ The predominance of a specific clone, masking minority clones with a lower CCF, and the arbitrariness of the biopsies in the primary tissue, may not reflect the set of genomic aberrations contained in the clones that make up the tumor bulk, being able to have a significant greater discrepancy between the primary tissue and the ctDNA profile.^24^

Next, the authors inquired the capability of predicting relapse from serial timepoints of plasma during follow-up. A progressive increase in VAF in relapsed patients was observed, indicating that MRD cannot be immediately detected in plasma after surgery, but can be successfully captured with ctDNA sampling at follow-up.^8^ The detection of longitudinal ctDNA can be used to identify residual disease following patients’ standard primary treatment and is associated with poorer PFS across different tumor types. Moreover, many of these observational studies have demonstrated that the detection of ctDNA typically precedes radiological relapse by a median lead time of 3-8 months.^8^, ^9^, ^10^, ^11^ However, it remains to be elucidated whether interventional clinical trials during surveillance before radiological relapse will impact on survival outcomes. Ongoing trials in patients with triple-negative breast cancer (cTRAK TN, NCT03145961) are investigating the role of ctDNA detection during follow-up and the safety and activity of pembrolizumab in treating MRD.

Bonazzi et al.^17^ demonstrated that ctDNA variants can be detected in patients with EAC and has prognostic value for survival. However, the use of ctDNA as a predictive marker in patients with EAC is still limited. To develop a successful ctDNA-guided strategy in patients with GEA, more sensitive and specific a assays would be needed before using them as a tool in interventional trials. Another question to be addressed in future studies is whether we need to personalize or not ctDNA assays to monitor MRD.
